# Association of genetic variants in *TPMT*, *ITPA*, and *NUDT15* with azathioprine-induced myelosuppression in southwest china patients with autoimmune hepatitis

**DOI:** 10.1038/s41598-021-87095-0

**Published:** 2021-04-12

**Authors:** Qiang Miao, Lin Yan, Yanhong Zhou, Yi Li, Yuangao Zou, Lanlan Wang, Yangjuan Bai, Junlong Zhang

**Affiliations:** Department of Laboratory Medicine/Research Center of Clinical Laboratory Medicine, West China Hospital of Sichuan University, No.37, Guoxue Xiang, Wuhou District, Chengdu, 610041 China

**Keywords:** Genetics, Immunology, Biomarkers, Molecular medicine, Rheumatology

## Abstract

This study aimed to investigate the influence of TPMT*3C, ITPA, NUDT15, and 6-thioguanine nucleotides (6-TGN) on azathioprine (AZA)-induced myelosuppression in Southwest China patients with autoimmune hepatitis (AIH). A total of 113 Chinese patients with AIH receiving AZA maintenance treatment were evaluated. The relevant clinical data of the patients were collected from the hospital information system. Genotyping of TPMT*3C(rs1142345), ITPA (rs1127354) and NUDT15(rs116855232) was conducted using a TaqMan double fluorescent probe. The concentration of 6-TGN was determined using UPLC-MS/MS. Among AIH patients treated with AZA, 40 (35.4%) exhibited different degrees of myelosuppression. The *NUDT15* variant was associated with leukopenia (*P* = 8.26 × 10^–7^; OR = 7.5; 95% CI 3.08–18.3) and neutropenia (*P* = 3.54 × 10^–6^; OR = 8.05; 95% CI 2.96–21.9); however, no significant association with myelosuppression was observed for TPMT*3C and *ITPA* variants (*P* > 0.05). There was no significant difference in 6-TGN concentration between AIH patients with or without myelosuppression (*P* = 0.556), nor was there a significant difference between patients with variant alleles of TPMT*3C, ITPA, or *NUDT15* and wild-type patients (*P* > 0.05). Interestingly, it was found that patients with a lower BMI had higher adjusted 6-TGN levels and a higher incidence of myelosuppression (*P* = 0.026 and 0.003). This study confirmed that *NUDT15* variants are a potential independent risk predictor for AZA-induced leukopenia and neutropenia. BMI may be a crucial non-genetic factor that affects the concentration of AZA metabolites and myelosuppression. In addition, the 6-TGN concentration in red blood cells does not reflect the toxicity of AZA treatment, and new biomarkers for AZA therapeutic drug monitoring need further research.

## Introduction

Azathioprine (AZA) is a prodrug of thiopurine that has been used as a classic immunosuppressant in the clinical treatment of autoimmune diseases for more than 60 years. The autoimmune hepatitis (AIH) guidelines issued by the European Hepatology Society in 2015 propose combination therapy with prednisone and AZA as the first-line program for the induction of remission and maintenance therapy for AIH patients^1^. However, the effects of AZA medication vary significantly between individuals, and about 15% of patients experience adverse drug reactions that lead to treatment interruption^2^. Among these, the most common and severe adverse reaction in the early stage of treatment is myelosuppression; patients are usually asymptomatic, but the risk of developing a life-threatening is significantly increased^3^.

Individual differences in AZA treatment are closely related to its metabolism in the body. As a prodrug, AZA has no biological activity. 6-Thioguanine nucleotides (6-TGN) and the methylation products 6-methylmercaptopurine ribonucleotides (6-MMPr)^4,5^ are the final active metabolites, that cause the risk of myelosuppression and liver toxicity. The metabolic process of AZA is complex because it involves various enzymes and is affected by gene polymorphisms. Thiopurine methyltransferase (TPMT) is a crucial enzyme involved in AZA metabolism. TPMT gene mutation leads to a decrease or deletion of TPMT activity, which affects the balance between the active metabolites 6-MMPr and 6-TGN. Patients with gene mutations or low enzymatic activity tend to have an increased concentration of 6-TGN, making them prone to myelosuppression^6^. However, the risk allele frequency of TPMT*3C T>*C* is low (1.3%) in East Asian populations^7^. Therefore, the *TPMT* genotype testing and mercaptopurine dosage guidelines issued in European and American countries do not apply to Asian and Chinese populations because they cannot fully explain the low tolerable dose of AZA in the Asian population and the high incidence of adverse reactions.

Inosine triphosphate pyrophosphatase (ITPA) is widely present in various organs and tissues, including red blood cells. ITPA catalyses the hydrolysis of inosine triphosphate (ITP) into inosine monophosphate (IMP) and protects cells from DNA damage induced by accumulated non-canonical nucleotides. The hydrolysis of ITP to IMP catalyzed by ITPA still occurs during AZA metabolism. The incidence of the *ITPA 94C*>*A* mutation in the Asian population is as high as 14–19%^8^. Studies have shown that the *ITPA* genotype can explain and predict the resistance and side effects of thiopurine therapy and affect treatment outcomes^9,10^. A 2014 study on thiopurine-related leukopenia in patients with inflammatory bowel disease (IBD) found that the *NUDT15 c.415C*>*T* gene mutation is closely related to AZA-induced leukopenia^11^. Subsequent studies in Japan, China and India^12–15^ all found that the *NUDT15 c.415C*>*T* gene polymorphism is closely related to AZA-induced leukopenia. In patients with acute lymphoblastic leukaemia, especially in Asian population, it has also been found that *NUDT15 c.415C*>*T* gene mutations may be related to thiopurines tolerance and myelosuppression^16,17^. AZA is more widely applied in other autoimmune diseases, especially AIH, than in IBD. However, there are only two related reports for AIH patients, including a case report^18,19^. There has not been a comprehensive assessment of the relationship between AZA toxicity and genetic variants of TPMT*3C T>*C*, *ITPA 94C*>*A,* and *NUDT15 c.415C*>*T* in patients with AIH in Southwest China.

The primary purpose of the present study was to investigate the relationship between TPMT*3C T>*C*, *ITPA 94C*>*A* and *NUDT15 c.415C*>*T* single nucleotide variants and AZA-induced myelosuppression in patients with AIH. In addition, influence of red blood cell 6-TGN levels on myelosuppression was explored in patients with AIH to clarify the value of thiopurine metabolite detection in guiding drug-dose adjustment in the treatment of such patients.

## Materials and methods

### Subjects

In this study, a total of 113 patients with AIH who had received AZA maintenance treatment from September 2017 to September 2019 at West China Hospital of Sichuan University were included. The inclusion criteria included patients who were clinically diagnosed with AIH and received AZA treatment for more than 12 weeks, followed up regularly in the hospital and aged 18 years or older. Exclusion criteria included patients under the age of 18, those with a recent history of blood transfusion or administered medications that may lead to myelosuppression, those experiencing pregnancy and lactation, those with an incomplete medical history, or those who were not regularly followed up. The relevant clinical data were collected from the hospital information system (HIS), including sex, age, height, weight, dosage of medication, and regular follow-up to monitor the results of routine blood tests. This study was performed in accordance with the Declaration of Helsinki and was approved by the Ethics Committee of West China Hospital of Sichuan University. Written informed consent was obtained from all enrolled patients.

### Treatment and toxicity

Base on the instructions, the initial dose of AZA treatment for AIH patients was based on body weight, usually 1.0–1.5 mg kg^−1^ per day. Complete blood cell count (CBC) was performed weekly for the first month after the beginning of treatment and every 2 weeks for the subsequent 2 months. After three months of treatment or at the time point when AZA toxicity occurred, blood samples were collected from the patients for measurement of the 6-TGN concentration and genetic testing. The primary time endpoint of follow-up was 12 weeks, and the secondary time endpoint was the occurrence of myelosuppression, withdrawal, or switching to other drugs. Patients who developed myelosuppression during treatment monitoring after starting AZA had their dose reduced first, usually to 50% of the initial dose. If abnormal haematological indicators related to myelosuppression did not subside, AZA was discontinued and other drugs were administered. The clinician responsible for the treatment decided to reduce the drug dose and discontinued the drug, if necessary.

According to the World Health Organization standards for acute and subacute toxicity of anticancer drugs^20^, myelosuppression is defined as a white blood cell (WBC) count of < 4 × 10^9^/L, platelet (PLT)count of < 100 × 10^9^/L, or neutrophil (NEU) count of < 2 × 10^9^/L. The relevant haematological indicators gradually decreased by more than 50% during the treatment monitoring period for patients with mild myelosuppression (WBC, 3.0–3.9 × 10^9^/L, PLT, 75–99 × 10^9^/L, NEU, 1.5–1.9 × 10^9^/L) compared with those before treatment. After a comprehensive assessment by the clinician in charge of treatment, AZA-induced myelosuppression was considered when other diseases that cause myelosuppression were excluded.

### Gene analysis

Total genomic DNA was extracted from peripheral blood using the YAOJINBAO® DNA purification kit (Beijing Sino-Era Gene Tech Co. Ltd, China) according to the manufacturer's instructions. TPMT*3C T>*C(rs1142345)*, *ITPA 94C*>*A(rs1127354)* and *NUDT15 c.415C*>*T (rs116855232)* genotyping was performed using liquid-phase molecular hybridisation SNP genotyping technology with a TaqMan double fluorescent probe. The reagent used for the genotype detection was the SNP analysis reagent Yaojinfen® (Beijing China Times Gene Co., Ltd.), and the detection instrument was a Fluotec 48E Trace fluorescence detector (Xi'an TianLong Science and Technology Co. Ltd). The detection process was divided into two steps: melting and hybridisation. The melting temperature was 95 °C, and the optimum hybridisation temperatures for TPMT*3C, *ITPA* and *NUDT15* were 58 °C, 64 °C and 60 °C, respectively. The standard test procedure involved 55 cycles, and the total reaction time was generally within 2.5 h. Both negative and positive controls were included in all sample analysis processes to ensure the authenticity of the results.

### Determination of the 6-TGN concentration

The concentration of 6-TGN, an active metabolite of AZA, was determined using our previously published ultra-performance liquid chromatography-tandem mass spectrometry (UPLC-MS/MS) method^21^. The results, expressed in pmol/8 × 10^8^ RBC, were similar to those of a previous study.

### Statistical analysis

Statistical analysis was performed using IBM SPSS software (version 23.0; SPSS Inc., Chicago, IL, USA). The Hardy–Weinberg equilibrium (HWE) was calculated for each polymorphism. Equilibrium was indicated by *P* > 0.05 (chi-squared statistics). Continuous data were summarised using the medians and interquartile ranges (IQR) and compared using the Kruskal–Wallis *H*-test or Mann–Whitney *U*-test. Categorical variables were reported as frequencies and percentages, and Pearson chi-square tests or Fisher's exact tests were performed to analyse the differences between two independent groups. The odds ratio (OR) and 95% confidence interval of the allele model were determined using logistic regression analysis. All statistical tests were two-tailed, and *P* < 0.05 was deemed significant.

## Results

### Patient characteristics

Based on the inclusion and exclusion criteria, 113 eligible patients were included in the study. Most of them were female (n = 97, 85.8%), and the female to male ratio was about 6:1. Their ages were in the range of 26–77 years. The characteristics of the patients are summarised in Table 1. Ultimately, 40 (35.4%) patients exhibited different degrees of myelosuppression. Age, sex, weight, smoking, initial dose of AZA, liver function indicators, baseline WBC count, PLT count, and NEU count were not significantly different between individuals with or without myelosuppression (*P* > 0.05). Patients with myelosuppression had a lower height than those without myelosuppression (1.56 m vs. 1.60 m, *P* = 0.018). There were also significant differences in the distribution of body mass index (BMI) between the two groups (*P* = 0.003). The proportion of patients with a BMI of less than 18.5 kg/m^2^ in the myelosuppression group was 15%, whereas the proportion of patients in the group without myelosuppression was 0% (Table 1).

**Table 1:** Baseline characteristics of included subjects [median(IQR)].

Clinical features	With myelosuppression (N = 40)	Without myelosuppression (N = 73)	*P*
Age (years)	52.0 (45.2, 60.8)	50.0 (43.0, 57.0)	0.168
Female/male	36/4	61/12	0.348
Height (m)	1.56 (1.52, 1.60)	1.60 (1.55, 1.62)	0.018*
Weight (kg)	56.0 (50.5, 63.0)	59.0 (52.8, 65.0)	0.127
BMI (kg/m^2^)	23.3 (20.6, 24.9)	23.1 (21.4, 24.7)	0.714
< 18.5	15.0% (6/40)	0% (0/73)	0.003*
18.5 ≤ BMI ≤ 24	52.5% (21/40)	63.0% (46/73)	
> 24	32.5% (13/40)	37.0% (27/73)	
AZA dose (mg kg^−1^ d^−1^)	1.07 (0.85, 1.27)	0.98 (0.86, 1.44)	0.978
WBC_0^†^ (10^9^/L)	6.55 (5.24, 7.91)	6.53 (5.29, 7.52)	0.570
PLT _0^†^ (10^9^/L)	274 (183, 344)	238 (176, 338)	0.583
NEU_0^†^ (10^9^/L)	3.25 (2.71, 4.24)	3.40(2.79, 4.35)	0.606
ALT(IU/L)	122.5 (82.5, 147.8)	120.8 (77.7, 152.6)	0.881
AST(IU/L)	128.4 (97.6, 154.7)	127.3 (99.1, 168.4)	0.652
ALP(IU/L)	172.6 (120.6, 222.4)	187.9 (140.2, 214.5)	0.631
GGT(IU/L)	180.3 (120.2, 236.5)	166.4 (81.1, 248.3)	0.517
TPMT*3C TT/TC/CC	40/0/0	71/2/0	0.539
*ITPA 94C*>*A CC/CA/AA*	27/13/0	50/22/1	0.741
*NUDT15 c.415C*>*T CC/CT/TT*	26/11/3	62/11/0	0.012*
Smoke Yes/No	0/40	5/68	0.159

*BMI* body mass index, *AZA* azathioprine, *WBC* white blood cell count, *PLT* platelet count, *NEU* neutrophil count, *ALT* alanine aminotransferase, *AST* aspartate aminotransferase, *ALP* alkaline phosphatase, *GGT* glutamyl transferase.

^†^Represents the results prior to azathioprine treatment.

*Significant (*P* < 0.05).

The TPMT*3C, ITPA and *NUDT15* genotype distributions were in Hardy–Weinberg equilibrium among the included populations (*P* = 1.00, *P* = 0.53 and *P* = 0.822). The detailed distributions are shown in Table 2. No TPMT*3C (T>*C)* homozygote (CC) was detected in the study; two cases were heterozygotes (TC, 1.8%), and the remaining 111 cases were wild-type (TT, 98.2%). The C and T allele frequencies were 0.9% and 99.1%, respectively. Among the 113 analysed individuals, 77 patients were *ITPA 94C*>*A* wild-type (CC, 68.1%), 35 patients were heterozygotes (CA, 31.0%), and only one subject was homozygous (AA, 0.9%). The frequencies of C and A alleles were 83.6% and 16.4%, respectively. In the same cohort, the number of subjects displaying *NUDT15 c.415(C*>*T)* genotypes CC, CT, and TT were 88 (77.8%), 22 (19.5%), and 3 (2.7%), respectively. The frequency of the variant allele T was 12.4%. There were significant differences in the genotype distribution of *NUDT15 c.415(C*>*T)* between individuals with and without myelosuppression (*P* = 0.012), whereas no significant difference was observed in the distribution of TPMT*3C (T>*C)* and *ITPA 94C*>*A* genotypes (*P* < 0.05, Table 1).

**Table 2:** Allele distribution of *NUDT15 c.415C*>*T, ITPA 94C*>A and TPMT*3C genotypes.

Gene	Genotype	N	Genotype frequency (%)	Allelic association		
				Allele	Allele frequency (%)	HWE *P*-value
TPMT*3C T>*C(rs1142345)*	TT	111	98.2	T	99.1	1.00
	TC	2	1.8	C	0.9	
*IPTA94C*>*A (rs1127354)*	CC	77	68.1	C	83.6	0.53
	CA	35	31.0	A	16.4	
	AA	1	0.9			
*NUDT15 c.415C*>*T (rs116855232)*	CC	88	77.8	C	87.6	0.822
	CT	22	19.5	T	12.4	
	TT	3	2.7			

### Association of phenotype with myelosuppression

Among the 113 patients, 47(41.6%) carried at least one variant allele in TPMT*3C (rs1142345), ITPAc.94C>*A (rs1127354)*, or *NUDT15c.415C*>*T (rs116855232)*, and 66 (58.4%) were wild-type allele carriers. No significant differences were observed in age, BMI, AZA dose, 6-TGN concentration, and adjusted 6-TGN concentration between the individuals in the variation and wild-type groups (*P* > 0.05, Table 3). Myelosuppression indicators were compared between the two groups at the 12th week or when adverse events occurred. It was found that the median levels of WBC_12w and NEU_12w in the variation group were 4.99 (4.03, 6.66) × 10^9^/L and 2.82 (2.21, 4.04) × 10^9^/L, respectively, whereas those in the wild-type group were 6.39 (4.64,7.72) × 10^9^/L and 3.67 (2.75, 5.22) × 10^9^/L, respectively; there were significant differences between the two groups (*P* = 0.003 and 0.002, respectively), but the median level of PLT_12w was not significantly different(*P* > 0.05, Table 3). There was also no significant differences in the incidence of myelosuppression between the variation and wild-type groups (*P* > 0.05, Table 3).

**Table 3:** Analysis of related indicexes between patients with genetic variation and wild-type patients [median(IQR)].

	Variation group^†^ (n = 47)	Wild-type group (n = 66)	*P*
Age (years)	54 (45, 62)	50 (43, 54)	0.063
BMI (kg/m^2^)	22.5 (20.6, 24.2)	23.2 (21.7, 26.1)	0.229
AZA dose (mg kg^−1^ day^−1^)	0.95 (0.85, 1.18)	1.08 (0.87, 1.47)	0.083
6-TGN (pmol/8 × 10^8^RBC)	125.29 (87.43, 237.21)	115.15 (64.16, 196.81)	0.262
Adjusted 6-TGN^‡^ (pmol/8 × 10^8^RBC mg kg day)	0.037 (0.021, 0.066)	0.036 (0.019, 0.062)	0.958
WBC_12w^§^ (10^9^/L)	4.99 (4.03, 6.66)	6.39 (4.64,7.72)	0.003**
PLT_12w^§^ (10^9^/L)	122 (89, 220)	140 (96,196)	0.942
NEU_12w^§^ (10^9^/L)	2.82 (2.21, 4.04)	3.67 (2.75, 5.22)	0.002**
**Myelosuppression**			0.346
Yes	19 (40.4%)	21 (31.8%)	
No	28 (59.6%)	45 (68.2%)	

*BMI* body mass index, *AZA* azathioprine, *6-TGN* 6-thioguanine nucleotides, *WBC* white blood cell count, *PLT* platelet count, *NEU* neutrophil count, *RBC* red blood cell.

^†^At least one variant allele in TPMT*3C (rs1142345), ITPAc.94C>*A (rs1127354)* or *NUDT15c.415C*>*T (rs116855232)*.

^‡^6-TGN concentration adjusted by body weight and daily dose.

^§^12 weeks from the start of AZA treatment or the time when adverse events occurred.

**Significant (*P* < 0.01).

### Association of genotypes with myelosuppression

The relationship between myelosuppression-related indicators and TPMT*3C T>*C(rs1142345)*, *ITPA94C*>*A(rs1127354)*, and *NUDT15c.415C*>*T (rs116855232)* genotypes was further analysed. Detailed results are presented in Table 4. Of the 111 patients with wild-type (TT) *TPMT∗ 3C*, 15 (13.5%) had leukopenia, 34 (30.6%) had thrombocytopenia, and 10 (9.0%) had neutropenia. Two heterozygotes (TC) did not develop myelosuppression, but their AZA dosage was less than 1 mg kg^−1^ day^−1^. There was no significant correlation between the TPMT*3C genotype and AZA-induced leukopenia, thrombocytopenia, or neutropenia (*P* = 0.747, 0.351 and 0.658, respectively). Among 77 patients with wild-type (CC) *ITPA 94C*>*A*, the number of patients exhibiting leukopenia, thrombocytopenia, and neutropenia was seven (9.1%), 24 (31.2%) and three (8.6%), respectively. Among the 35 heterozygous (CA) patients, eight (22.9%) patients developed leukopenia, 10 (28.6%) developed thrombocytopenia, and three (8.6%) developed neutropenia. Only one patient was homozygous for AA. This patient did not develop myelosuppression, and his AZA dose was less than 1 mg kg^−1^ day^−1^. Compared with wild-type (CC), patients with variant allele A (CA + AA) had no significant difference in the risk of leukopenia, thrombocytopenia, and neutropenia (*P* = 0.102, 0.657, and 0.862, respectively).

**Table 4:** Association of myelosuppression with *NUDT15 c.415C*>*T, ITPA 94C*>A and TPMT*3C genotypes.

	TPMT*3C T>*C(rs1142345)*		Allele model OR (95% CI) *P*-value	*IPTA94C*>*A (rs1127354)*			Allele model OR (95% CI) *P*-value	*NUDT15 c.415C*>*T (rs116855232)*			Allele model OR (95% CI) *P*-value
	TT (n = 111)	TC (n = 2)		CC (n = 77)	CA (n = 35)	AA (n = 1)		CC (n = 88)	CT (n = 22)	TT (n = 3)	
**Leukopenia**											
Yes	15 (13.5%)	0 (0%)	1.05(1.03–1.07) 0.747	7 (9.1%)	8 (22.9%)	0 (0%)	2.09 (0.85–5.15) 0.102	6 (6.7%)	6 (27.3%)	3 (100%)	7.5 (3.08–18.3) 8.26 × 10^–7^***
No	96 (86.5%)	2 (100%)		70 (90.9%)	27 (77.1%)	1 (100%)		82 (93.2%)	16 (72.7%)	0 (0%)	
**Thrombopenia**											
Yes	34 (30.6%)	0 (0%)	1.43 (1.31–1.56) 0.351	24 (31.2%)	10 (28.6%)	0 (0%)	0.84 (0.38–1.38) 0.657	25 (28.4%)	8 (36.4%)	1 (33.3%)	1.34 (0.58–3.08) 0.488
No	77 (69.4%)	2 (100%)		53 (68.8%)	25 (71.4%)	1 (100%)		63 (71.6%)	14 (63.6%)	2 (66.7%)	
**Neutropenia**											
Yes	10 (9.0%)	0 (0%)	1.09 (1.05–1.14) 0.658	7 (9.1%)	3 (8.6%)	0 (0%)	0.89 (0.25–3.22) 0.862	3 (3.4%)	5 (22.7%)	2 (66.7%)	8.05 (2.96–21.9) 3.54 × 10^–6^***
No	101 (91.0%)	2 (100%)		70 (90.9%)	32 (91.4%)	1 (100%)		85 (96.6%)	17 (77.3%)	1 (33.3%)	

*CI* confidence interval.

***Significant (*P* < 0.001).

When evaluated according to *NUDT15c.415C*>*T* genotype, AZA-induced leukopenia was observed in six (6.7%) patients with the CC allele and six (27.3%) patients with the CT allele. Three (100%) patients with the homozygote allele (TT) had leukopenia. Leukopenia was significantly associated with the *NUDT15c.415C*>*T* variant allele T (*P* = 8.26 × 10^–7^; OR = 7.5; 95% CI 3.08–18.3; Table 4). The incidence of neutropenia in patients with CC, CT and TT genotypes was 3.4% (3/88), 22.7% (5/22), and 66.7% (2/3), respectively. Compared with patients carrying the wild-type genotype (CC), those carrying variant allele T (CT + TT) had a much higher risk of developing neutropenia (*P* = 3.54 × 10^–6^; OR = 8.05; 95% CI 2.96–21.9; Table 4). The positive predictive values of *NUDT15* variant allele T for leukopenia and neutropenia were 36% (9/25) and 28% (7/25), respectively. However, there was no significant difference in the incidence of thrombocytopenia among patients with different genotypes (*P* = 0.488). Two (66.7%) of the homozygous (TT) patients were treated with an AZA dosage less than 1 mg kg^−1^ day^−1^.

### Relationship between 6-TGN concentration and myelosuppression

In 113 patients, the concentration of 6-TGN ranged from 2.07 to 2554.09 pmol/8 × 10^8^ RBC, and the median (interquartile range) concentration was 123.34 (79.89, 231.77) pmol/8 × 10^8^ RBC. The corrected concentration of 6-TGN ranged from 0.001 to 0.416 pmol/8 × 10^8^ RBC· mg kg day, and the median (interquartile range) concentration was 0.036 (0.021, 0.066) pmol/8 × 10^8^ RBC·mg kg day. The concentration of 6-TGN was analysed among the different variable groups. Detailed results are presented in Supplementary Table S1. In general, the levels of 6-TGN and adjusted 6-TGN were not significantly different between patients with and without myelosuppression (*P* = 0.556 and 0.876, respectively), and these levels also did not differ significantly according to sex or AZA dose group (*P* > 0.05). In addition, there was no obvious correlation between the levels of 6-TGN and adjusted 6-TGN among different genotypes of TPMT*3C T>*C*, *ITPA94C*>*A* and *NUDT15c.415C*>*T* (*P* > 0.05). However, it was found that BMI may have affected the adjusted 6-TGN level in this study population (*P* = 0.026).

## Discussion

AZA is a classic maintenance treatment drug for AIH. Such drugs affect individuals differently and produce severe adverse reactions, which have received widespread attention. Although the most recent Clinical Pharmacogenetics Implementation Consortium (CPIC) publication includes dosing guidelines based on *TPMT* and *NUDT15* metabolizer status^22^, they mainly apply to AZA treatment in ALL and IBD patients. Due to variation by race and type of disease, and the lack of pharmacogenetic data for AZA treatment in patients with AIH, there is no clear guideline for establishing therapeutic dosing regimen and metabolite adequate concentration standards for AZA treatment of Chinese patients with AIH. This study found that patients with at least one genetic variant in TPMT*3C, ITPA94C>*A*, and *NUDT15c.415C*>*T* had lower WBC and NEU counts than those of the wild-type patients. T allele variation in *NUDT15c.415C*>*T* was an independent risk factor for leukopenia and neutropenia.

The blood toxicity of AZA is related to genetic polymorphisms in *TPMT*. Mutation or low enzyme activity leads to a high concentration of 6-TGN, which increases the risk of myelosuppression^6^. Therefore, in 2005, the FDA began including the pre-administration *TPMT* genotype test on AZA's drug label^23^. In AZA pharmacogenomics research, TPMT (*2, *3A and *3C) is the most widely studied single nucleotide polymorphism for AZA metabolism to date. The incidence of *TPMT* allelic variants is approximately 10–15% in the Caucasian population (commonly TPMT*3A)^24^. However, the incidence of *TPMT* allelic variants in the Chinese population is lower than that in the Caucasian population. The literature reports that the *TPMT* allele (commonly TPMT*3C) accounts for less than 5% prevalence, which is close to that of Japan and South Korea^25,26^. In this study, the mutation frequency of the C allele in TPMT*3C was observed to be 0.9%. There were only two (1.8%) patients with heterozygous mutations of TPMT*3C, and neither of them exhibited myelosuppression; however, among TPMT*3C wild-type allele carriers, 15 (13.5%) patients suffered leukopenia, 34 (30.6%) suffered thrombocytopenia, and 10 (9.0%) experienced neutropenia. The above results indicate that the TPMT*3C gene test has limited predictive value for AZA-induced myelosuppression in the Chinese population. Therefore, although TPMT*3C has been considered the leading risk factor for AZA-induced myelosuppression, no significant difference was observed in this study, which may be due to its low prevalence and the small sample size of this study.

*ITPA* polymorphism is another essential enzyme involved in the metabolism of AZA. Studies have shown that the *ITPA c.94C*>*A* mutation can cause the enzyme activity to decrease, causing the toxic metabolite 6-TITP to accumulate in the body, and produce flu-like symptoms, gastrointestinal reactions, skin rash, pancreatitis, and even neutropenia and liver damage, which ultimately lead to interruption of treatment^9,27^. The incidence of *ITPA 94C*>*A* mutations observed in this study was 16.4%, which is consistent with that of other studies^8^. Only one case of homozygous mutation was observed among 113 subjects. Since this case received an AZA dose < 1 mg kg^−1^ day^−1^, myelosuppression was not observed. In this study, the incidence of leukopenia and thrombocytopenia in patients with heterozygous mutations was 22.9% (8/35) and 28.6% (10/35), respectively. However, a significant differences was not observed between different genotypes of *ITPA 94C*>*A* regarding AZA-induced leukopenia, thrombocytopenia, and neutropenia. This may be because *ITPA 94C*>*A* is mainly related to AZA-induced liver toxicity. Alternatively, the small sample size could be the reason for the insignificant difference.

NUDT15 belongs to the Nudix (nucleoside diphosphate linked to x) hydrolase superfamily. It mainly consists of pyrophosphohydrolase, which acts on nucleoside diphosphates linked to other moieties. NUDT15 hydrolyses 6-thio-GTP (TGTP) and 6-thio-GDP (TGDP) into 6-thio-GMP (TGMP), which reduces their cytotoxic effects. Mutation of *NUDT15* increases the cytotoxicity of mercaptopurine drugs. Most studies have shown that the incidence of *NUDT15* allelic mutations in the Asian populations is 8.5–16%^28,29^, whereas it is less than 1% in Caucasian populations^30^. The frequency of NUDT15 mutations in IBD patients in Japan and South Korea is 12% and 10.4%, respectively, but it can be as high as 32.1% in Chinese patients with autoimmune diseases^31^. The frequency observed in this study was 12.4%, which is similar to the frequency of 9.4% in patients with AIH reported by Xiaoli Fan et al.^19^ Recent studies have found that *NUDT15 c.415C*>*T* variants were associated with thiopurine-induced leukopenia, particularly in Asian populations^28,32–34^. In 2014, Yang et al.^11^ revealed that *NUDT15 c.415C*>*T* allelic mutation is significantly associated with AZA-induced leukopenia in Korean IBD patients (*P* = 5.58 × 10^−43^, OR = 8.61). Subsequently, it was also confirmed in Japanese IBD patients that *NUDT15 c.415C* > *T* allelic mutation is closely related to AZA-induced early leukopenia (*P* = 1.92 × 10^−16^, OR = 28.4)^13^. Studies by Xiang Fei et al.^31^ and Xiaoli Fan et al.^19^ on Chinese autoimmune diseases and AIH patients also showed that *NUDT15 c.415C*>*T* SNP is significantly related to AZA-induced early leukopenia (*P* = 1.79 × 10^−7^; OR = 7.59 and *P* < 0.00001; OR = 20.41, respectively). The present results were concordant with those of previous studies, which showed that the *NUDT15 c.415C*>*T* allelic mutation was associated with early leukopenia (*P* = 8.26 × 10^−7^; OR = 7.5). It was also found that the *NUDT15 c.415C*>*T* mutation is implicated in AZA-induced myelosuppression with neutropenia as the primary manifestation (*P* = 3.54 × 10^−6^; OR = 8.05). Therefore, compared with TPMT*3C and *ITPA 94C*>*A*, the detection of *NUDT15 c.415C*>*T* in the Chinese population may have better predictive value for AZA-induced myelosuppression with leukopenia and neutropenia as the primary manifestations. In this study, the predictability of the *NUDT15* variant allele for leukopenia was 36%, which is lower than the value of 42.3% reported by Xiaoli Fan et al.^19^ Schaeffeler et al. observed that the *NUDT15* variant contributed to 13% of AZA-induced leukopenia among Caucasian people; further, they observed that in combination, *TPMT* and *NUDT15* variants explain ~ 50% of myelosuppression among AZA users of European descent^35^. This shows that multi-gene analysis may have better predictive value for AZA-induced leukopenia. Moreover, research has shown that *NUDT15 c.415C*>*T* was associated with not only early (< 8 weeks) leukopenia but also middle (8–24 weeks) and late (> 24 weeks) leukopenia^14^. However, these findings could not be fully confirmed in the present study because of the shorter follow-up duration (12 weeks), which is a limitation of this study.

It is well known that 6-TGN is the active metabolite responsible for AZA efficacy and cytotoxicity, and one of the side effects of AZA therapy is myelosuppression. Therapeutic drug monitoring (TDM) of one of the pharmacologically active metabolites of thiopurines, 6-TGN, has proven beneficial^36^. However, there is no unified conclusion about the relationship between the concentration of 6-TGN in red blood cells and adverse reactions. Asada et al.^12^ and Xiang Fei et al.^31^ observed no statistically significant difference in concentration between different *NUDT15c.415C*>*T* genotypes. However, Xiaoli Fan et al.^19^ reported that the 6-TGN concentration in CT genotype patients with *NUDT15c.415C*>*T* variants was significantly higher than that in CC wild-type allele carriers. The above studies showed no significant difference in the concentration of 6-TNG between patients with and without leukopenia. This finding was replicated in the present study (*P* = 0.556, Table S1). Among the 113 AIH patients included in this study, significant differences in 6-TGN concentration and adjusted 6-TGN concentration were not observed among the different genotypes of TPMT*3C, ITPA 94C>*A* and *NUDT15c.415 C*>*T* (*P* > 0.05, Table S1). The same is true between the different gender groups and maintenance dose groups. However, significant differences were found in the adjusted concentration of 6-TGN between patients with different BMIs (*P* = 0.026, Table S1). In addition, an analysis of baseline characteristics revealed that patients with lower height and lower BMI had a higher incidence of myelosuppression. This indicates that BMI may be a crucial non-genetic factor affecting the concentration of AZA active metabolites and myelosuppression. For drugs with a complex metabolism and narrow therapeutic index, such as AZA, future individualised drug research may integrate genetic variation factors and more clinical data into a standard scoring model, fully considering how each factor affects patient risk. In recent years, studies have proposed that measuring the concentration of peripheral blood mononuclear cells (PBMCs) for immunosuppressants could be useful as a valuable biomarker to improve TDM^37^. In consideration of this and the results of the present study, it can be determined that the new therapeutic monitoring method for detecting AZA metabolites may have more clinical value in PBMCs than in whole blood.

One shortcoming is that this was a single-centre study with a limited number of patients and regional limitations, which preclude adequate statistical inference. In addition, the follow-up time was short, and the long-term adverse reactions could not be thoroughly evaluated. Finally, commercial kits were used to detect the most common mutations in the Asian population. The lack of comprehensive testing of AZA metabolism-related genes may have led to biased results.

## Conclusion

In conclusion, the present study confirmed that genetic variants of *NUDT15 c.415 C*>*T* were associated with AZA-induced myelosuppression in Southwest Chinese patients with AIH, and they represent a potential independent risk predictor that leads to leukopenia and neutropenia. It was also found that patients with a lower height and BMI had a higher frequency of myelosuppression. In addition, the 6-TGN concentration in red blood cells does not reflect the efficacy and toxicity of AZA treatment. Hence, new biomarkers for AZA therapeutic drug monitoring need to be explored further.

## Supplementary Information

Supplementary Information.
