# Supplementary material for: Association of genetic variants in TPMT, ITPA, and NUDT15 with azathioprine-induced myelosuppression in southwest china patients with autoimmune hepatitis
Source: Sci Rep. 2021 Apr 12;11:7984. doi: 10.1038/s41598-021-87095-0 (PMC8042108; doi:10.1038/s41598-021-87095-0)
Supplement: Supplementary file 1 — Supplementary Information. [file 41598_2021_87095_MOESM1_ESM.docx]

**Association of Genetic Variants in *TPMT, ITPA,*and *NUDT15*With Azathioprine-Induced Myelosuppression in Southwest China patients with Autoimmune Hepatitis**

| **Table S1 6-TGN and adjusted 6-TGN concentrations in groups with different characteristics [Median(IQR)]** | | | | | |
| --- | --- | --- | --- | --- | --- |
| Characteristics | N | 6-TGN  (pmol/8×10^8^RBC) | *P* | Adjusted 6-TGN^†^ (pmol/8×10^8^RBC· mg·kg·d) | *P* |
| Gender |  |  | 0.41 |  | 0.097 |
| female | 88 | 130.4 (79.9, 234.4) |  | 0.039 (0.022, 0.068) |  |
| male | 25 | 97.4 (79.1, 216.1) |  | 0.025 (0.014, 0.055) |  |
| AZA dose |  |  | 0.057 |  | 0.171 |
| ≤1.0 mg^.^kg^-1.^d^-1^ | 51 | 113.9 (62.4, 197.8) |  | 0.028 (0.017, 0.062) |  |
| >1.0 mg^.^kg^-1.^d^-1^ | 62 | 143.9 (88.1, 270.1) |  | 0.042 (0.024, 0.071) |  |
| BMI (kg/m^2^) |  |  | 0.704 |  | 0.026* |
| <18.5 | 6 | 174.7 (101.3, 236.5) |  | 0.083 (0.045, 0.093) |  |
| 18.5≤BMI≤24 | 67 | 130.3 (79.7, 216.6) |  | 0.044 (0.21, 0.067) |  |
| >24 | 40 | 100.2 (76.7, 252.6) |  | 0.028 (0.014, 0.053) |  |
| Myelosuppression |  |  | 0.556 |  | 0.876 |
| Yes | 40 | 126.8 (85.6, 232.7) |  | 0.035 (0.02, 0.084) |  |
| No | 73 | 116.1 (67.6, 228.8) |  | 0.036 (0.021, 0.064) |  |
| *NUDT15c.415C>T (rs116855232)* |  |  | 0.064 |  | 0.227 |
| CC | 88 | 106.6 (54.1, 165.2) |  | 0.036 (0.015, 0.052) |  |
| CT+TT | 25 | 131 (86.2, 235.3) |  | 0.038 (0.021, 0.07) |  |
| *TPMT*3C T>C(rs1142345)* |  |  | 0.896 |  | 0.836 |
| TT | 111 | 123.3 (80.1, 230.1) |  | 0.036 (0.021, 0.065) |  |
| TC | 2 | 512.3 (-) |  | 0.167 (-) |  |
| *ITPA94C>A(rs1127354)* |  |  | 0.299 |  | 0.603 |
| CC | 77 | 110.8 (64.6, 193.4) |  | 0.035 (0.021, 0.085) |  |
| CA+AA | 36 | 127.2 (85.2, 239.1) |  | 0.039 (0.021, 0.065) |  |
| Abbreviations: BMI, body mass index; AZA, azathioprine; 6-TGN, 6-thioguanine nucleotides。  ^†^ 6-TGN concentration adjusted by body weight and the daily dose. | | | | | |

Qiang Miao, Lin Yan, Yanhong Zhou, Yi Li, Yuangao Zou, Lanlan Wang, Yangjuan Bai^#^, Junlong Zhang^#^
